# Allele-Specific Impairment of *GJB2* Expression by *GJB6* Deletion del(*GJB6*-D13S1854)

**DOI:** 10.1371/journal.pone.0021665

**Published:** 2011-06-29

**Authors:** Juan Rodriguez-Paris, Marta L. Tamayo, Nancy Gelvez, Iris Schrijver

**Affiliations:** 1 Department of Pathology, Stanford University School of Medicine, Stanford, California, United States of America; 2 Instituto de Genética Humana, Universidad Javeriana, Bogotá, Colombia; 3 Fundación Oftalmológica Nacional, Bogotá, Colombia; 4 Department of Pediatrics, Stanford University School of Medicine, Stanford, California, United States of America; University Hospital Hamburg-Eppendorf, Germany

## Abstract

Mutations in the *GJB2* gene, which encodes connexin 26, are a frequent cause of congenital non-syndromic sensorineural hearing loss. Two large deletions, del(*GJB6*-D13S1830) and del(*GJB6*-D13S1854), which truncate *GJB6* (connexin 30), cause hearing loss in individuals homozygous, or compound heterozygous for these deletions or one such deletion and a mutation in *GJB2*. Recently, we have demonstrated that the del(*GJB6*-D13S1830) deletion contributes to hearing loss due to an allele-specific lack of *GJB2* mRNA expression and not as a result of digenic inheritance, as was postulated earlier. In the current study we investigated the smaller del(*GJB6*-D13S1854) deletion, which disrupts the expression of *GJB2* at the transcriptional level in a manner similar to the more common del(*GJB6*-D13S1830) deletion. Interestingly, in the presence of this deletion, *GJB2* expression remains minimally but reproducibly present. The relative allele-specific expression of *GJB2* was assessed by reverse-transcriptase PCR and restriction digestions in three probands who were compound heterozygous for a *GJB2* mutation and del(*GJB6*-D13S1854). Each individual carried a different sequence variant in *GJB2*. All three individuals expressed the mutated *GJB2* allele in *trans* with del(*GJB6*-D13S1854), but expression of the *GJB2* allele in c*is* with the deletion was almost absent. Our study clearly corroborates the hypothesis that the del(*GJB6*-D13S1854), similar to the larger and more common del(*GJB6*-D13S1830), removes (a) putative *cis*-regulatory element(s) upstream of *GJB6* and narrows down the region of location.

## Introduction

**Figure 1:**
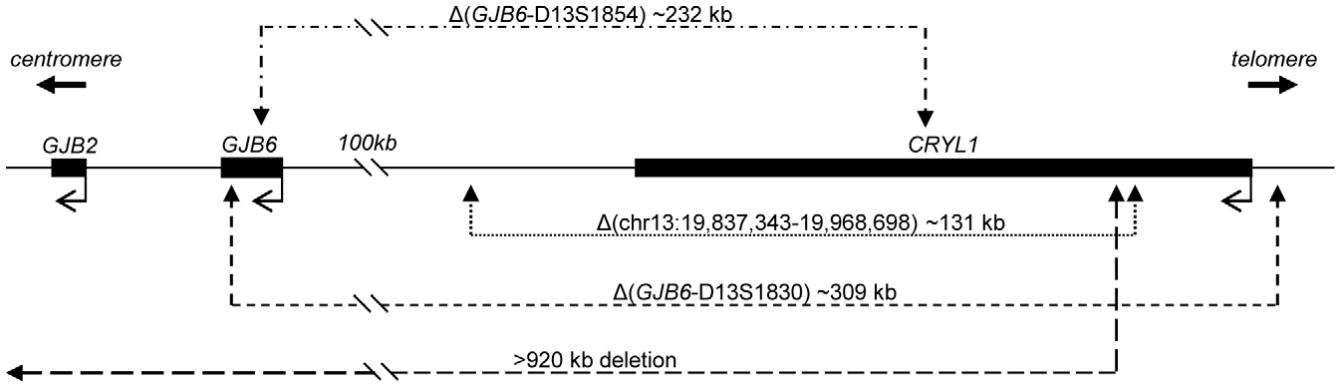
Map of the region on chromosome 13q11-12 disrupted by deletions. The ∼365 kb DNA section encompasses three genes (dark boxes), the breakpoints of the deletions (arrowheads) and their range (dashed lines). All elements are drawn approximately to scale. *GJB6* and *CRYL1* are impacted by three of the four deletions; however *GJB2* is directly affected only by the largest deletion. del(*GJB6*-D13S1854) and del(*GJB6*-D13S1830) are investigated in this study. The transcriptional start sites are shown by bent arrows.

The DFNB1 locus at chromosome 13q11-q12 includes the *GJB2* and *GJB6* genes, which respectively encode connexin 26 (Cx26) and connexin 30 (Cx30). These connexin proteins are co-expressed and co-localized in the cochlea, where they create heteromeric gap junctions [1] and make important contributions to cochlear homeostasis [2]. *GJB2* mutations are the most common genetic etiology of prelingual non-syndromic sensorineural hearing loss [3], [4]. Even though many recessive *GJB2* mutations have been described (http://davinci.crg.es/deafness/), so far only four recessive mutations affecting *GJB6* or the region upstream have been reported, all of which are deletions [5]–[12] (Fig 1). The most common are del(*GJB6*-D13S1830) and del(*GJB6*-D13S1854), which truncate the *GJB6* gene [5]–[9]. The other two are private mutations, one of which (>930 kb) deletes both *GJB2* and *GJB6* [11] and the other (del(chr13:19,837,343–19,968,698) does not affect either gene and is located upstream of *GJB6* [10], [12] (Fig 1).

We have recently reported that the larger of the two most common deletions del(*GJB6*-D13S1830), disrupts human *GJB2* expression at the transcriptional level in a allele-specific manner [13], presumably by removing one or more *cis*-regulatory elements located within the deleted region, which are as yet obscure. Given the large proportion (up to ∼13% in the USA [14]) of individuals left with only one recessive *GJB2* mutation after genetic testing instead of the two expected, it seems plausible that some of the unidentified mutations could be *cis*-regulatory in nature and would be located outside the transcribed region.

The goal of this study was to determine whether the smaller and less common del(*GJB6*-D13S1854) deletion, similar to del(*GJB6*-D13S1830), disrupts human *GJB2* expression at the transcriptional level, which would lend further support to the putative presence of regulatory element(s) upstream of *GJB6*. It has been assumed that del(*GJB6*-D13S1854) and del(*GJB6*-D13S1830) would affect *GJB2* expression similarly, however until now this has never been demonstrated. Our study also sought to narrow down the region were the supposed *cis*-regulatory element(s) may be located, based on the fact that del(*GJB6*-D13S1854) is ∼77 kb smaller than del(*GJB6*-D13S1830) and that it is included within that deletion. In order to investigate the relative abundance of *GJB2* transcripts from each allele, allele-specific analyses based on reverse-transcriptase PCR (RT-PCR) were conducted with RNA extracted from buccal epithelial cells, which are known to express both Cx26 and Cx30 [10], [13]. In all three probands, each of whom carried a different sequence variant in *GJB2,* the expression of *GJB2* from the chromosome bearing the del(*GJB6*-D13S1854) deletion was virtually absent (Table 1).

**Table 1:** Genotypes and phenotypes of study subjects.

Proband	*GJB2* variant(s)	*GJB6* variant(s)	Phenotype
CE1	WT/WT	Δ(*GJB6*-D13S1830)/Δ(*GJB6*-D13S1830)	Profound hearing loss
CE2	S199F/WT	Δ(*GJB6*-D13S1830)/WT	Profound hearing loss
CE3	S199F/WT	Δ(*GJB6*-D13S1854)/WT	Profound hearing loss
CE4	Q80P/WT	Δ(*GJB6*-D13S1854)/WT	Severe hearing loss
CE5	35delG/WT	Δ(*GJB6*-D13S1854)/WT	Moderate to Severe hearing loss
**Control**	***GJB2*** ** variant(s)**	***GJB6*** ** variant(s)**	**Phenotype**
CTR1	WT/WT	WT/WT	None
CTR2	S199F/WT	WT/WT	None
CTR3	S199F/WT	WT/WT	None

## Materials and Methods

### Study subjects

Three controls with neither del(*GJB6*-D13S1854) nor del(*GJB6*-D13S1830) and five probands were included in this study. Three of the affected individuals were of Colombian origin (CE1, CE2 and CE3) as were two of the controls (CTR2 and CTR3). One proband (CE1) was homozygous for del(*GJB6*-D13S1830), one carried del(*GJB6*-D13S1830) and a *GJB2* mutation in *trans* (CE2), and three were compound heterozygous for del(*GJB6*-D13S1854) and a *GJB2* mutation in *trans* (CE3, CE4 and CE5). This study was approved by the Stanford University institutional review board, and written informed consents were obtained from all participants (Table 1). The participating probands had hearing loss ranging from moderate to profound. Control CTR1 had no identified sequence changes; the other two controls were related to probands and were heterozygous carriers of the *GJB2* Ser199Phe (596C>T) mutation. Control CTR2 was the father of proband CE2 and control CTR3 was the mother of proband CE3. None of the controls were affected. Direct sequencing and a restriction fragment length polymorphism (PCR-RFLP) assay identified or confirmed all sequence changes.

### RT-PCR

**Figure 2:**
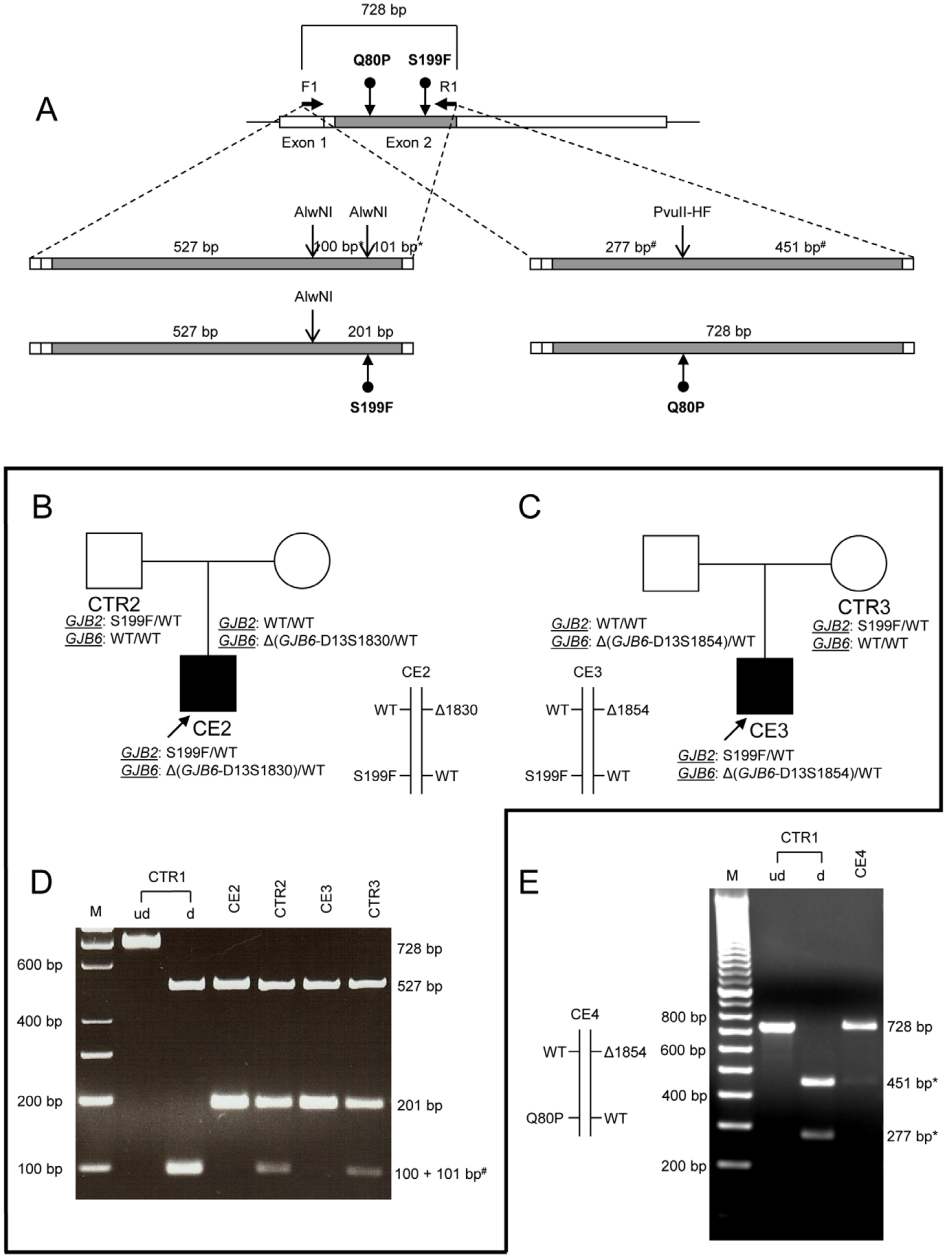
Allele-specific expression analysis for *GJB2*, based on Ser199Phe (596C>T) and Gln80Pro (239A>C). (A) Schematic design of AlwNI and PvulI-HF digestion analysis. A 728 bp *GJB2* cDNA amplicon was digested with AlwNI to distinguish Ser199Phe (S199F) from the wild-type allele, or with PvulI-HF to distinguish Gln80Pro (Q80P). Bars indicate *GJB2* exons. The translated region within exon 2 is shaded. The relative positions of the *GJB2* sequence changes (dotted arrows) and restriction sites (arrows) are indicated. Digestion fragment sizes are listed for both sequence changes and the wild-type. (^#^) and (*) indicate fragments unique to the wild-type alleles in *cis* with del(*GJB6*-D13S1830) or del(*GJB6*-D13S1854), respectively. (B) Pedigree of proband CE2, who carries del(*GJB6*-D13S1830) in *trans* with Ser199Phe in *GJB2*. (C) Pedigree of proband CE3, who carries the del(*GJB6*-D13S1854) deletion in *trans* with Ser199Phe in *GJB2*. (D) *GJB2* expression analysis based on the Ser199Phe sequence variant. The Ser199Phe mutation prevents AlwNI from digesting the original 201 bp fragment into a 101 and a 100 bp fragment, as illustrated in A. The digested 100 and 101 bp bands represent the wild-type allele and are undetectable in proband CE2 and almost entirely absent (the very faint remaining band is not visible on the printed picture) in proband CE3, indicating a complete lack of *GJB2* expression from the del(*GJB6*-D13S1830) allele and an almost a complete lack from the del(*GJB6*-D13S1854) carrying chromosome. (E) Allele-specific expression analysis for *GJB2*, based on Gln80Pro (239A>C). This mutation prevents PvulI-HF from digesting the 728 bp RT-PCR product in proband CE4. The digested bands represent the wild-type allele, which are almost undetectable in proband CE4 indicating a nearly complete lack of *GJB2* expression from the del(*GJB6*-D13S1854) carrying chromosome. M  =  marker; ud  =  undigested, no restriction enzyme added; d  =  digested, restriction enzyme added.

**Figure 3:**
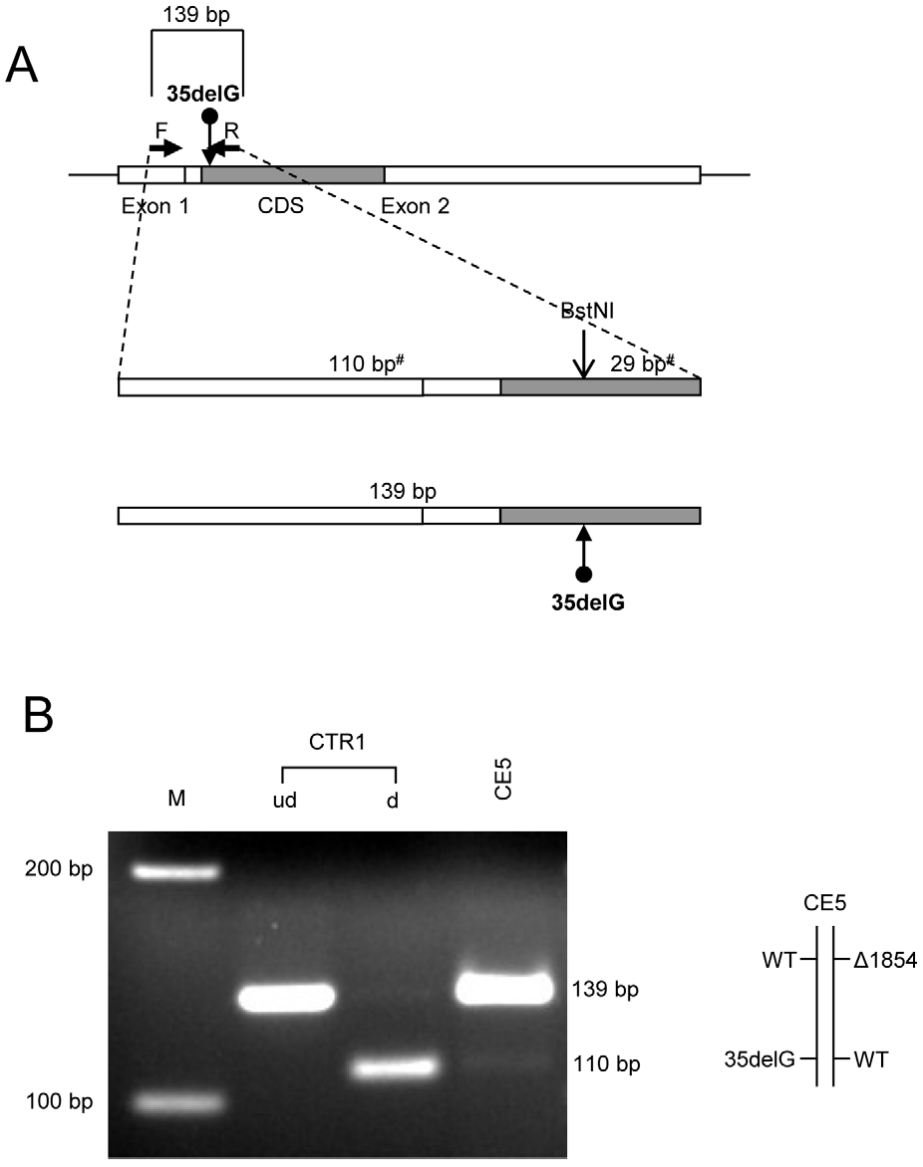
Allele-specific expression analysis for *GJB2*, based on 35delG. (A) A schematic representation (not to scale) of BstNI digestion analysis [10]. A 139 bp PCR product was amplified from *GJB2* cDNA. Bars indicate *GJB2* exons. The translated region within exon 2 is shaded. *GJB2* mutation 35 delG is indicated (dotted arrows), as is the restriction site (arrow). The mutation eliminates a single BstNI restriction enzyme site. (B) BstNI digestion of the product amplified from proband CE5 yields an undigested 139 bp band from the 35delG allele, but the 110 bp digestion product expected from the wild-type allele is virtually absent. The wild-type control (CTR1) yields the expected 110 bp band. M =  marker; ud  =  undigested, no restriction enzyme added; d  =  digested, restriction enzyme added.

Total RNA was isolated from buccal epithelium cells as previously described [13]. Briefly, cells were collected on Cytosoft® Plus brushes (CooperSurgical, Trumbull, CT) and immediately submerged in RNA*later*® (Ambion, Austin, TX). Cells were spun down and total RNA was extracted following the RNAeasy mini kit protocol (Qiagen, Valencia, CA). cDNA synthesis was performed according to the SuperScript III (Invitrogen, Carlsbad, CA) reverse transcription method and a negative control to rule out gDNA contamination was included for each sample. The negative controls followed all procedure steps with the matching samples and remained negative (data not shown). We used cDNA-specific primers to avoid amplification from gDNA. First, a forward primer in *GJB2* exon 1 was paired with a reverse primer in exon 2 to generate a 728 bp PCR product from the cDNA template only [13], [15]. These primers amplify a region enclosing the whole *GJB2* coding sequence (CDS) (Table 2, Fig 2A). ß-actin primers specific for cDNA [16] were used as an RNA expression control for the sample homozygous for the del(*GJB6*-D13S1830) deletion (CE1, Table 1). Second, for evaluation in the presence of the 35delG mutation we used primers designed by Wilch *et al*. and Wilcox *et al*. [10], [17], which were located in exons 1 and 2, as well. These primers generated a smaller (139 bp) PCR product, also however specific only to cDNA (Table 2, Fig 3A). Four µl cDNA per 20 µl reaction and 40 amplification cycles were used for all the PCR amplifications described above.

**Table 2:** PCR-RFLP used to distinguish *GJB2* variants Gln80Pro (239A>C), Ser199Phe (596C>T) and 35delG.

Mutation	Primer and sequence (5′–3′)	Product size(bp)	Restriction enzyme	Fragment sizes(bp)
Q80P	aF1: TTCCTCCCGACGCAGAGCAA ^b^R1: GGGCAATGCGTTAAACTGGC	728	PvuII-HF	Wt: 451+277Mt: 728
S199F	aF1: TTCCTCCCGACGCAGAGCAA ^b^R1: GGGCAATGCGTTAAACTGGC	728	AlwNI	Wt: 527+101+100Mt: 527+201
35delG	^c^F: CGCAGAGACCCCAACGCCGAGA ^d^R: GCTGGTGGAGTGTTTGTTCACACCCGC^e^	139	BstNI	Wt: 110+29Mt: 139

a Rodriguez-Paris *et al.* [13]. ^b^Mueller *et al*. [15]. ^c^Wilch *et al*. [10]. ^d^Wilcox *et al*. [17]. ^e^Underscored base is mismatched to eliminate BstNI restriction enzyme site. Wt  =  wild-type; Mt  =  Mutant.

### Allele-specific assays

Relative transcript abundance from each *GJB2* allele was estimated by RT-PCR and RFLP assays. In the instance of *GJB2* change Gln80Pro (239A>C), the 728 bp amplicon from the *GJB2* CDS was digested with PvuII-HF to avoid star activity (the digestion of sequences which are similar but not identical to the restriction enzyme's unique recognition sequence). The variant eliminates a single PvuII-HF restriction site, which yields an undigested product (728 bp) instead of two fragments (451 and 277 bp) when this variant is absent (Table 2 and Fig 2A). Variant Ser199Phe (596C>T) eliminates an AlwNI restriction enzyme site, which limits digestion of the Ser199Phe allele product to two fragments (527 bp and 201 bp), instead of three (527, 101 and 100 bp) for the wild-type allele (Table 2 and Fig 2A).

For the 35delG mutation, a mismatched reverse primer [10] eliminates a BstNI restriction site from the 139 bp RT-PCR product, and this allows differentiation between the mutant and wild-type alleles. The digested wild-type allele is associated with fragment sizes of 110 bp and 29 bp, whereas the amplicon associated with the mutant allele remains undigested (Table 2 and Fig 3A).

### Restriction enzyme digestion

Digestion was performed as previously described [13]. In brief, 10 µL of PCR product was digested with 10 units of the applicable restriction enzyme for two hours, according to the manufacturer's protocol (New England Biolabs, Beverly, MA). Conditions conducive to star activity were avoided and high fidelity restriction enzymes were chosen when available. Products were electrophoresed on 3% agarose gels or on 3.5% NuSieve 3∶1 agarose gels (Lonza, Rockland, ME).

### Sequence analysis of the basal promoter, exon 1 and the *GJB2* coding region

The basal promoter and exon 1 of the *GJB2* gene of three selected samples (CE4, CE5 and CTR1) were investigated for the presence of mutations according to Matos *et al.* [18]. In brief, a 1009 bp *GJB2* segment was amplified which included part of the 5′ UTR, exon 1 and the donor splice site of intron 1. A 539 bp segment from this amplicon was sequenced using a hemi-nested forward primer and the same reverse primer. This sequenced segment included the 128 bp proximal promoter region, exon 1 and its donor splice site. The *GJB2* CDS, the flanking sequence of intron 1 (including the acceptor splice site) and a fraction of the 3′UTR were also sequenced for all participants.

## Results

**Figure 4:**
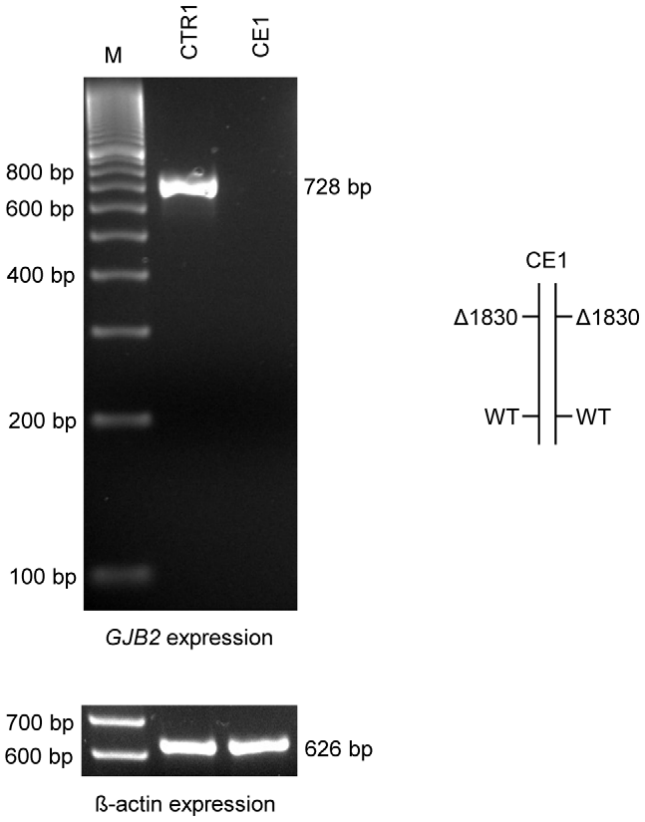
*GJB2* expression of a del(*GJB6*-D13S1830) homozygous patient. PCR amplification was performed after reverse transcription using primers specific for *GJB2* cDNA (F1 and R1, Table 2 and Fig 2A). The wild-type control CTR1 amplified a 728 bp segment characteristic for *GJB2* while del(*GJB6*-D13S1830) homozygous patient CE1 did not express that band at all. ß-actin was used as an RNA expression control. Both samples amplified the expected 626 bp ß-actin band specific for cDNA.

We determined the allele-specific expression of *GJB2* in four study subjects who were compound heterozygous for either del(*GJB6*-D13S1854) or del(*GJB6*-D13S1830) and a mutation in *GJB2*, in *trans* (Table 1). At least one parent of each proband was genotyped for both *GJB2* and *GJB6* to confirm that the mutations were indeed present on opposite alleles. We also analyzed the allele-specific expression of *GJB2* in a fifth individual (CE1) who was homozygous for the del(*GJB6*-D13S1830) deletion. Based on our previous results [13], this individual with profound hearing loss was expected to not express *GJB2* at all. The complete failure to amplify the 728 bp segment from the *GJB2* cDNA indeed indicates the complete absence of *GJB2* expression from both del(*GJB6*-D13S1830) alleles (Fig 4). *GJB2* expression from an unaffected individual with no identified mutations in either *GJB6* or *GJB2* (CTR1) was used as a control and the 728 bp segment was amplified as expected (Fig 4). cDNA-specific ß-actin expression [16] was used for RNA expression control. Both proband and control amplified the expected 626 bp segment, confirming normal ß-actin expression (Fig 4).

Study participant CE2 inherited the del(*GJB6*-D13S1830) deletion from his mother and the Ser199Phe mutation in *GJB2* from his father (Fig 2B). In order to make possible the distinction between the affected and wild-type *GJB2* alleles, we performed allele-specific restriction digestions. The complete and reproducible absence of the 100 and 101 bp bands associated with the wild-type allele for Ser199Phe (CE2, Fig 2D) demonstrated a lack of *GJB2* expression from the del(*GJB6*-D13S1830) allele in this proband. The presence of the 201 bp fragment associated with a Ser199Phe allele confirms expression of the opposite *GJB2* allele (CE2, Fig 2D). Allele-specific expression results from an individual with no detectable mutations in either *GJB6* or *GJB2* (CTR1) and from the father of the proband who is a carrier of Ser199Phe (CTR2) were used as controls and were amplified as well as digested simultaneously with the sample from the proband (Fig 2D). Both controls expressed the expected alleles.

To determine whether del(*GJB6*-D13S1854) has the same effect on *GJB2* expression as del(*GJB6*-D13S1830), we analyzed the allele-specific expression of *GJB2* in an unrelated individual who carried the same *GJB2* (Ser199Phe) mutation, with the smaller *GJB6* deletion (del(*GJB6*-D13S1854) ) in *trans*. Proband CE3 inherited the deletion from his father and the Ser199Phe mutation from his mother (Fig 2C). Interestingly, in this case we detected minimal residual expression of the wild-type *GJB2* allele in *cis* with the del(*GJB6*-D13S1854) deletion (not readily visible in the printed figure , Fig 2D). Both alleles are distinctly expressed by the mother of this proband (CTR3). All expression experiments were performed at least four times (twice each from two separate RNA preparations) and results were congruent.

To further elucidate whether these results reveal a consistent regulatory effect of the del(*GJB6*-D13S1854) mutation, we analyzed the allele-specific expression of *GJB2* in another two unrelated individuals who carried the same *GJB6* deletion with different *GJB2* mutations in *trans*. By sequence analysis of *GJB2*, proband CE4 was heterozygous for Gln80Pro (239A>C). The allele-specific expression assay demonstrated the 728 bp uncut band from that allele, but revealed only minimal residual expression at the wild-type fragments (CE4, Fig 2E). Thus, the *GJB2* transcript from the allele in *cis* with del(*GJB6*-D13S1854) is almost entirely absent (Fig 2E). The respective control in this case was a sample from an individual without *GJB2* mutations (CTR1), which clearly expressed only the expected wild-type allele. The third proband with del(*GJB6*-D13S1854) had moderate to severe hearing loss and was compound heterozygous with 35delG in the *GJB2* gene, in *trans*. Again, the allele-specific assay virtually lacked the 110 bp fragment associated with the wild-type allele, substantiating the almost complete down-regulation of *GJB2* expression from the chromosome with del(*GJB6*-D13S1854) (CE5, Fig 3B). The control (CTR1) on the other hand clearly exhibited the expected transcript.

We sequenced *GJB2* exon 1, its splice sites and the proximal promoter region in probands CE4 and CE5 as well as in control CTR1, to confirm that the allele-specific loss of *GJB2* expression was related to the *GJB6* deletions and not to another mutation on the same allele. No sequence changes were detected in the analyzed regions. Furthermore, all primer binding sites were investigated for potential sequence changes to minimize the possibility for bias in PCR amplification due to differences in primer binding affinity. No sequence changes in primer binding sites were identified.

## Discussion

The main purpose of this study was to demonstrate an effect on *GJB2* expression by del(*GJB6*-D13S1854), which is internal to the larger and more prevalent del(*GJB6*-D13S1830) deletion, which we studied previously [13]. Secondarily, we aimed to refine the location of the *GJB2* regulatory element(s) that is/are hypothesized to be within the deleted regions. Using immunostaining, a cell-specific loss of Cx26 expression was observed in the sweat glands of an individual compound heterozygous for 35delG in *GJB2* and del(*GJB6*-D13D1830) in *GJB6*. The functional effect on Cx26 protein pointed to the disruption of a putative *cis*-regulatory element which appears to function with cell-type specificity within the sweat gland [19]. Until recently [12], [13], however, no more direct support of the existence or effects of such elements was reported.

Deletion del(*GJB6*-D13S1854) is one of four reported DFNB1 deletions (Fig 1), of which only one (>920 kb deletion) directly affects the *GJB2* gene [11]. Another pathogenic DFNB1 deletion was identified entirely upstream from *GJB6* (Fig 1) in a large kindred of German extraction. This ∼131 kb deletion segregated with profound deafness when in *trans* with 35delG in *GJB2*. Allele-specific expression demonstrated markedly reduced expression for *GJB2* as well as *GJB6*, with under-representation of the allele with the deletion [10], [12]. This deletion remains unique to this family [12]. del(*GJB6*-D13S1854) is about 77 kb smaller than the ∼309 kb del(*GJB6*-D13S1830), which includes the former. It has been assumed but not previously demonstrated that del(*GJB6*-D13S1854) impairs *GJB2* expression in an allele-specific manner similar to what has been reported for del(*GJB6*-D13S1830). We determined the effect on *GJB2* expression by using qualitative allele-specific RT-PCR analyses to assess the relative abundance of *GJB2* transcript from both alleles. The studies were performed on readily accessible buccal epithelium, which expresses both Cx26 and Cx30 and has been postulated to contain one or more functional regulatory elements that control expression of both proteins [10], [12], [13]. Three probands were identified who carried del(*GJB6*-D13S1854) and each had different mutations in the *GJB2* gene, in *trans* (Table 1). Their hearing phenotypes ranged from moderate to profound and were not obviously correlated with the severity of the *GJB2* change (Table 1). In contrast to the previously reported and additional, currently included, individuals with the larger del(*GJB6*-D13S1830) in whom we could not detect any *GJB2* expression from that allele (CE1 and CE2; [13]), there was reproducibly suppressed and barely detectable low residual *GJB2* expression from the chromosomes which carried del(*GJB6*-D13S1854) (CE3, CE4 and CE5, Figs 2 and 3). The controls expressed both alleles normally, as expected (CTR2 and CTR3, Fig 2D, and CTR1, Fig 3B). This interesting difference between the two deletions supports the putative presence of multiple regulatory elements upstream of *GJB6*. Because the smaller deletion maintains at least some residual *GJB2* expression one could assume that some such regulatory or locus-control elements are not deleted with the 77 kb shorter del(*GJB6*-D13S1854).

It is as yet unclear how, exactly, a lack of transcription results in a hearing loss phenotype and to what extent partial or complete deletion of the *GJB6* gene contributes to this phenotype in humans. However, some such processes have been studied in some of the connexins and are clinically relevant [20]. Co-regulation of the two connexins has been explored by immunohistochemistry as well as quantitative PCR analysis in Cx30 knock-out mouse cultures [21]. Cx26 mRNA and protein were down-regulated in non-sensory cochlear cells, which could be overcome by over-expression of Cx30 after transduction with bovine adeno-associated virus. The expression of Cx26 and Cx30 in mouse cultures was inter-related and reciprocal, because down-regulation of Cx26 in cultures of a mouse model with ablation of Cx26 negatively impacted the expression of Cx30 as well as the communication between cells [21].

In summary, we conclude that the hearing loss in probands with the del(*GJB6*-D13S1854) deletion (and a heterozygous mutation in *GJB2*) results from a lack of functional Cx26 protein similar to the patients bearing larger deletion del(*GJB6*-D13S1830) (Figs 2 and 4, [13]). Our results are supportive of the presence of *GJB2 cis*-regulatory element(s) upstream *GJB6* and narrow down the location of those putative element(s) that most powerfully impact *GJB2* expression.
